# Differential effects of wastewater treatment plant effluents on the antibiotic resistomes of diverse river habitats

**DOI:** 10.1038/s41396-023-01506-w

**Published:** 2023-09-08

**Authors:** Jangwoo Lee, Feng Ju, Karin Beck, Helmut Bürgmann

**Affiliations:** 1 Eawag, Swiss Federal Institute of Aquatic Science and Technology, 6047 Kastanienbaum, Switzerland; 2 Department of Environmental Systems Science, ETH Zurich, Swiss Federal Institute of Technology, Zurich, Switzerland; 3 Key Laboratory of Coastal Environment and Resources of Zhejiang Province, School of Engineering, Westlake University, 310030 Hangzhou, Zhejiang China; 4 Westlake Laboratory of Life Sciences and Biomedicine, 310024 Hangzhou, Zhejiang China; 5 Present Address: Departments of Microbiology, Immunology & Infectious Diseases, Cumming School of Medicine, and Biological Sciences, Faculty of Science, University of Calgary, Calgary, AB Canada

**Keywords:** Water microbiology, Antibiotics

## Abstract

Wastewater treatment plants (WWTPs) are key sources of antimicrobial resistance genes (ARGs) that could influence the resistomes of microbial communities in various habitats of the receiving river ecosystem. However, it is currently unknown which habitats are most impacted and whether ARGs, like certain chemical contaminants, could be accumulated or enriched in the river ecosystem. We conducted a systematic metagenomic survey on the antibiotic resistomes of WWTP effluent, four riverine habitats (water, suspended particles, sediment, epilithic biofilm), and freshwater amphipod gut microbiomes. The impact of WWTP effluent on the downstream habitats was assessed in nine Swiss rivers. While there were significant differences in resistomes across habitats, the wastewater resistome was more similar to the resistome of receiving river water than to the resistomes of other habitats, and river water was the habitat most strongly impacted by the WWTPs effluent. The sulfonamide, beta-lactam, and aminoglycoside resistance genes were among the most abundant ARGs in the WWTP effluents, and especially *aadA*, *sul1*, and class A beta-lactamase genes showed significantly increased abundance in the river water of downstream compared to upstream locations (*p* < 0.05). However, this was not the case for the sediment, biofilm, and amphipod gut habitats. Accordingly, evidence for accumulation or enrichment of ARGs through the riverine food web was not identified. Our study suggests that monitoring riverine antimicrobial resistance determinants could be conducted using “co-occurrence” of *aadA*, *sul1*, and class A beta-lactamase genes as an indicator of wastewater-related pollution and should focus on the water as the most affected habitat.

## Introduction

Antimicrobial resistance (AMR) has been recognized as an important threat to human public health, and the risks associated with infections with AMR pathogens have rapidly increased over the past decades partly due to misuse and overuse of antibiotics [1]. While epidemiological spread from human to human is important, AMR can also be transferred from animals and from environmental reservoirs to humans. The realization of the importance of these pathways has been conceptualized in the “One Health” perspective [2, 3] as the foundation of strategies to combat the spread of AMR. Therefore, it has become important to assess AMR risks not only in the traditional medical or clinical context, but also to study potential environmental reservoirs where AMR could reside and evolve and the routes by which they could eventually be transmitted back to humans or animals.

Switzerland harbors a large quantity of freshwater resources and the headwaters of many rivers which flow into other European countries. In the interest of its own population and for protection of its environment, but also due to its location upstream of many European rivers, Switzerland recognizes its responsibility to protect the water quality of Swiss Surface Waters. However, monitoring of water quality so far relies exclusively on traditional chemical, physical and microbiological parameters, but does not include AMR-related biomarkers. As AMR is increasingly recognized as a globally emerging environmental contaminant [4–6], developing a scientific basis for monitoring water quality of rivers also in terms of AMR is important.

It has now been well established that wastewater treatment plants (WWTPs) are major point sources of antibiotic resistance genes (ARGs) in rivers [7–10]. For instance, many studies reported that absolute abundances of ARGs in river water significantly increased after receiving WWTP effluents [10–13]. The impact of WWTPs is however not limited to water habitats. It has also been reported that relative abundances of ARGs downstream of wastewater discharge points are higher than in the upstream for biofilms [14–16] and sediments [12, 16–18], revealing that the impact of WWTPs can occur in various riverine habitats. However most previous studies relied on monitoring specific genetic targets or model organisms while broad resistome analyses over multiple habitats are lacking. Therefore, a systematic monitoring strategy for riverine AMR is needed to compare across multiple environmental habitats. To thoroughly evaluate this, resistomes of all the habitats need to be systematically profiled and compared with a unified approach.

Another key aspect that has not been studied so far, is the potential for dissemination or even accumulation of ARGs through the riverine food web, for example from a food source to the gut microbiome of an aquatic organism at a higher trophic level. In this study, the gut microbiota of freshwater amphipods (gammarids), which are among the most prevalent group of freshwater benthic macroinvertebrates, was chosen as an example. Freshwater amphipods feed on various freshwater microbes, including attached-growth forms such as epilithic biofilms [19, 20]. If aquatic microbiota and biofilms are significantly impacted by WWTP effluents, amphipod gut microbiomes could be affected as well, via resistant bacteria in their food source, or their mobile resistomes. Freshwater amphipods are themselves a food source for animals further up the food chain (e.g., predatory invertebrates and fish) and AMR determinants from effluent or other environmental sources could thus theoretically be passed further up the food chain.

To address the research needs discussed above and to provide an improved basis for future freshwater AMR monitoring efforts, we profiled antibiotic resistomes of 9 wastewater-receiving Swiss rivers from 165 metagenomes obtained from various environmental habitats. The studied habitats included two size fractions of river water (suspended particles with their particle-associated microbial community and free-living, planktonic microbes), sediment, and epilithic biofilm. We further sampled freshwater amphipods as representatives of low trophic level river fauna and analyzed their gut microbiome. The impact of WWTP effluent on the resistomes of these habitats was systematically assessed, and key underlying biological drivers were inferred. We test a number of key hypotheses: First, that WWTP effluent affects downstream resistomes in all habitats of the river, but that the degree of WWTP impact differs between habitats. Secondly, that the structure of microbial communities is an important determinant of the structure of the resistomes. Finally, we hypothesized that wastewater-born ARGs can be transferred to, accumulated, or enriched in the gut microbiome of low trophic level fauna (e.g., freshwater amphipods).

## Materials and methods

This study draws from the same sampling campaigns, and shares physio-chemical, and biological (16S rRNA gene amplicon sequencing) data with a parallel study based on a different culture- and phenotypic screening-based metagenomic approach (i.e., phenotypic metagenomics) [21]. In this work we address different research hypotheses and use additional data and different data analyses approaches, i.e., microbial community analysis using 16S rRNA gene amplicon sequencing data, and quantitative and statistical analysis of resistome and microbiome data.

### Field sampling and physicochemical analysis

**Fig. 1:**
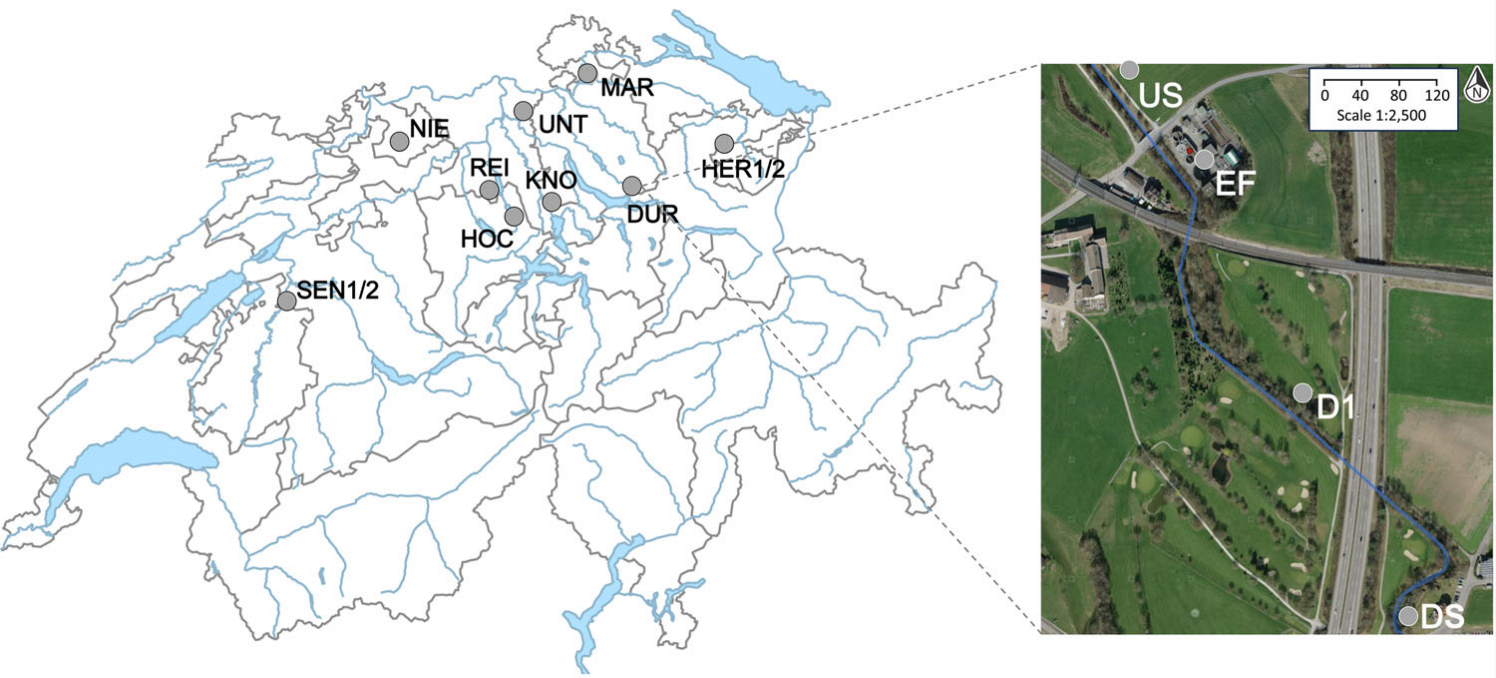
A total of 9 locations in Switzerland where WWTPs discharge into rivers were sampled in this study (left): Sensetal (SEN), Hochdorf (HOC), Weinland (MAR), Knonau (KNO), Reinach (REI), Duernten (DUR), Unterehrendingen (UNT), Niederdorf (NIE), and Herisau (HER). On the right, an example study site in the river Schwarz near Dürnten, Switzerland. US, EF, D1, and DS denote upstream, effluent, 130–1200 m downstream, and 800–1500 m downstream sampling locations, respectively. The aerial imagery for the inset (right) was obtained, and modified from map.geo.admin.ch.

Field sampling campaigns were performed at nine sampling sites encompassing Swiss WWTPs and the receiving rivers between July and October 2017 (Fig. 1): Sensetal (SEN), Hochdorf (HOC), Weinland (MAR), Knonau (KNO), Reinach (REI), Duernten (DUR), Unterehrendingen (UNT), Niederdorf (NIE), and Herisau (HER). For SEN and HER, sampling was performed twice, once under dry conditions (SEN2 and HER2), and once under conditions influenced by precipitation (SEN1 and HER1). Mild to moderate rainfall occurred also during or before three other samplings (SEN1, HOC, HER1). All other locations were sampled under dry or nearly dry (<0.3 mm/d in the previous 12 h) conditions. For each sampling campaign, river water, sediment, epilithic biofilms, and freshwater arthropod specimens were collected from the following locations: 130–1200 m upstream (US), 130–1200 m downstream (D1) and/or 800–1500 m downstream (DS) of the point of discharge of WWTP effluent. Wastewater effluents (EF) were obtained from the abovementioned WWTPs. All samples were stored at 4 °C in the dark, and transported to the laboratory within 12 h.

Physio-chemical parameters (water temperature, pH, DO = dissolved oxygen, and conductivity) were measured on-site using a portable multi-parameter probe (WTW Multi 3630 IDS, Xylem Analytics, Weilheim, Germany). In the laboratory, dissolved sulfate, and chloride anions in pre-filtered water sample aliquots were measured by ion chromatography as described in our previous studies [9, 12]. More details on the field sampling (e.g., site coordinates, sampling dates, receiving rivers, and precipitation) and physicochemical analsysis data were provided in the Methods and Supplementary Information of our previous study [21].

### Microbial biomass collection and DNA extraction

Biomass collection protocols are described in detail in our previous study [21]. In brief, particle-associated microbial biomass was collected by filtering water samples through 5.0 μm pore-size polycarbonate filters (TMTP14250, Merck Millipore, Darmstadt, Germany). Free-living (FL) planktonic microbial cells were obtained by filtering the 5.0 μm-filtered water samples through 0.22 μm pore-size polycarbonate filters (GTTP14250, Merck Millipore). The filtration volumes varied between 5–10 L depending on when filters clogged. To obtain biomass from sediments and epilithic biofilm slurries, samples were centrifuged at 16,000 × *g* for 5 min, and supernatants were removed. To obtain biomass from freshwater amphipod guts, an example of extracted amphipod gut materials is shown in Fig. S1.

DNA extraction was performed for the abovementioned biomass samples using appropriate extraction kits as described previously [21]. The DNeasy PowerWater Kit (Qiagen, Germany) and DNeasy PowerMax Soil Kit (Qiagen, Germany) were used for DNA extraction from water samples (filters) and sediments samples, respectively. The DNeasy PowerSoil Kit (Qiagen) was used for DNA extraction from both biofilm and amphipod gut pellet samples. DNA concentrations were measured using Qubit dsDNA HS Assay Kit (Thermo Fisher Scientific, Wilmington, DE, USA). A NanoDrop One spectrophotometer (Thermo Fisher) was used to characterize DNA concentration and purity (i.e., 260/280 and 260/230 ratios) in the extracts. The DNA concentrations and quality parameters were given in Supplementary Dataset S1.

### Sequencing and bioinformatic analysis

Shotgun metagenomic sequencing was performed on DNA extracts by a commercial sequencing company (Novogene, Hong Kong, China) using a HiSeq 4000 System (Illumina) with a paired-end (2 × 150 bp) strategy. bioinformatics analysis followed established procedures [17, 21]. Briefly, adapters were removed from raw reads, and quality filtering (removing the reads with >10 % ambiguous bases, or >50 % low quality bases) was performed. Then, read-based annotation of ARGs was performed using ARG-OAP v2.0 [22]. For further downstream analysis, we normalized to 16S rRNA gene abundance, i.e., gene copies per 16S rRNA gene (GP16S) [9], which will be hereafter referred to as relative abundance. The abundance tables of ARGs detected from the samples are shown in Dataset S2 (in terms of resistance subtype), and Dataset S3 (in terms of resistance class).

16S rRNA gene amplicon sequencing analysis was performed to explore the microbiomes of different habitats. Sequencing was performed by Microsynth (Switzerland) as described previously [21]. Particle-associated biomass samples were excluded from this analysis, for instance due to the relatively low DNA yield compared to free-living biomass. Barcode removal and quality trimming were performed using Illumina MiSeq Control Software v2.6 and Cutadapt v1.8 [23]. Further downstream analysis was performed using DADA2 according to the protocol modified from our previous manuscript [21], and referring to the work-flow suggested by the author of DADA2 v1.14.1 [24]. In short, (i) additional quality filtering of sequences was performed (truncQ = 2, and maxEE = 2), (ii) the error rates were inferred by DADA2 algorithms, (iii) the sample sequences were inferred using the previously derived error rates, iv) the paired ends were merged, and mis-merged chimeras were removed. The merged reads were defined as amplicon sequencing variants (ASVs), and these operational units (i.e., ASVs) were used when performing downstream statistical analysis of microbiomes. ASVs were normalized to the total number of reads obtained for each sample to obtain their relative abundance.

### Statistics and visualization

Kruskal–Wallis rank-sum test (a non-metric analysis of variance), and the post-hoc paired Wilcoxon signed-rank (i.e., paired test), or rank-sum test (i.e., non-paired test) (p-adjustment using the Benjamini–Hochberg method for multiple comparison) were applied to analyze significant differences of ARGs among different sampling locations (US, EF, D1, and DS) or different habitats. The signed-rank test was performed only when the sample sizes were equal, or similar to each other. The rank-sum test was used when the sample sizes among treatments were profoundly different from each other. Such tests were performed in terms of resistance class (i.e., the class of antibiotics to which the gene confers resistance) and/or subtype of ARGs. When implementing statistical tests in terms of class, classes with low abundances (i.e., bleomycin and carbomycin) were excluded. The following two R functions under the embedded package “stats v4.1.2” were used: kruskal.test(), and pairwise.wilcox.test (paired=TRUE for signed-rank; paired = FALSE for rank-sum test) with a default setting for treating missing (“NA”) values (i.e., na.action = ‘na.omit’) [25]. NMDS was performed to analyse the structural dissimilarities of resistomes and microbiomes, and Procrustes analysis was performed to analyse potential structural correlation between resistomes and microbiomes. One amphipod gut sample (i.e., GG04US) was identified as an extreme outlier from preliminary ordination results (data not shown), and excluded from the final NMDS and from all downstream analyses. Analysis of similarities (ANOSIM) was performed to test significant differences of ARG profiles between two habitats in terms of Bray–Curtis distance, and dispersion (i.e., distance to centroid) for each habitat was calculated to quantify variances of ARG profiles among sites within each habitat. All the multivariate statistical analyses were performed in R using the package “Vegan v2.5–7” [26]. Swiss map (Fig. 1, left) was produced using the R package “bfsMaps v0.9.6” [27], and heatmaps were produced using heatmap.2() in the R package “gplots v3.1.1” [28]. All the other graphics were realized using embedded R functions.

## Results and discussion

### Resistomes significantly differ among river habitats

To profile the Swiss river resistome comprehensively, 165 metagenomes with an average sequencing depth of 67 million reads (51–110 million) and 10 GB (7.6–16.5 GB) were generated (Dataset S1). Based on the annotation with ARG-OAP 2.0 against the SARG (Structured Antibiotic Resistance Gene) database [22, 29], we retrieved 677 ARG subtypes in 21 resistance classes from all habitats combined.

**Fig. 2:**
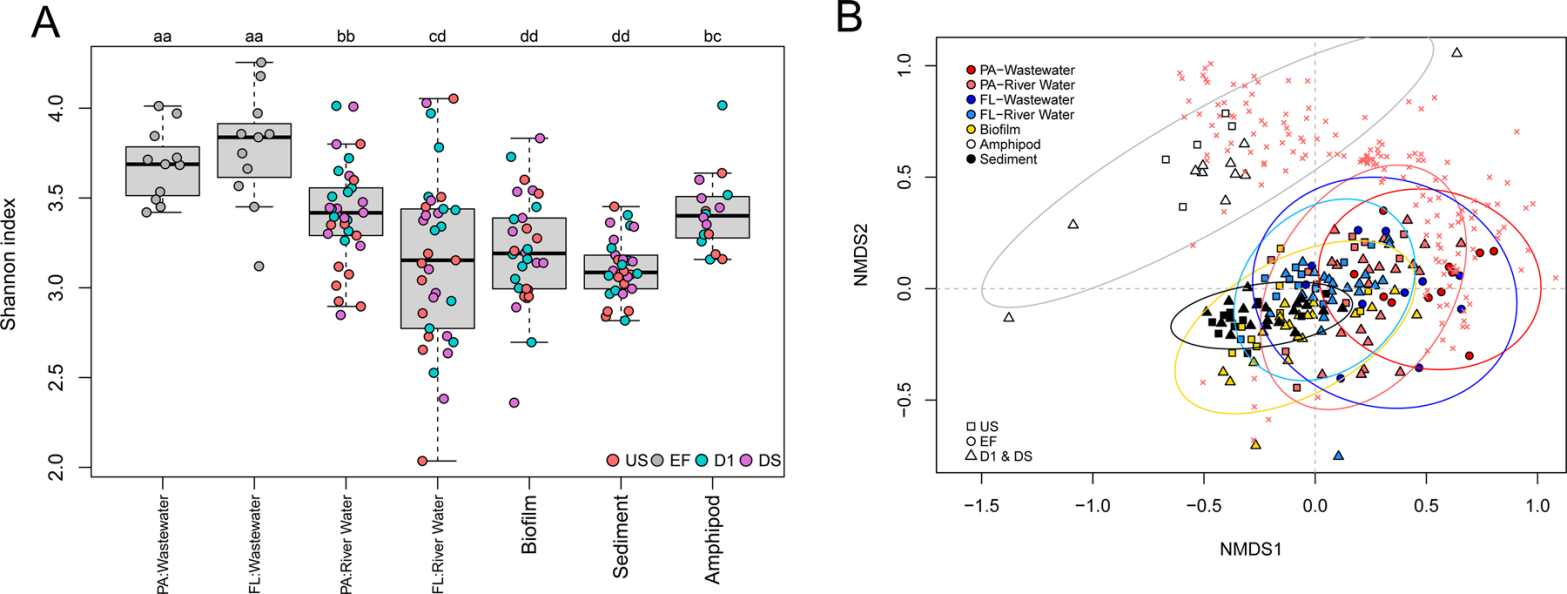
Alpha- and beta-diversity analysis of the resistomes of river habitats. **A** The Shannon index of ARGs in WWTP effluent and river habitats (FL: free-living fraction, PA: particle-associated fraction). Those habitats which share the same letter (i.e., a or b or c) are not statistically different from each other. **B** Non-metric multidimensional scaling (NMDS) analysis of the resistomes of various riverine compartments (waters, biofilms, sediments, and freshwater amphipod guts) and WWTP effluents (final stress = 0.168). The selected ARGs were scaled by the square root of *R*^2^. The variance ellipses using standard deviation of point scores (with confidence limits of 0.95) were highlighted in different colors for biofilm (in yellow), wastewaters (free-living in thick red; suspended particle in thick blue), river waters (free-living in light red; suspended particle in light blue), sediment (in black), and amphipod guts (in gray). The ARGs that are significantly correlated with the ordination (*p* ≤ 0.001) were displayed with the symbol × in red.

The alpha-diversity of ARGs measured by Shannon index was compared among WWTP effluent and different river habitats (i.e., particle-associated biomass and free-living bacteria in river water, biofilm, sediment, and amphipod gut). The Shannon index of ARGs for particle-associated biomass and amphipod guts were highest among river habitats, for instance significantly higher than for biofilm and sediment (Post-hoc Wilcoxon rank-sum tests; *p* < 0.05) (Fig. 2a). Furthermore, the Shannon index of ARGs in WWTP effluents was significantly higher than in other locations (US, D1, DS) for all the habitats (Fig. 2a). This suggests that habitats to some extent exert control over the diversity of ARGs. The variability of alpha-diversity was especially high in river water. The Shannon index of ARGs in downstream D1 sites was significantly higher than in US sites for particle-associated bacteria in river water (*p* < 0.05), but not for the other habitats. More comprehensive analysis on the impact of effluents in terms of collective and individual ARG abundances will be provided in sections “River water resistomes were significantly impacted by wastewater effluents” and “No consistent effects of wastewater effluent on non-water habitats”.

Structural dissimilarities among resistomes of different habitats were evaluated using NMDS of ARG relative abundance data (Dataset S2). The habitats grouped into overlapping but distinct clusters in the ordination (Fig. 2b). Significant differences were confirmed by ANOSIM analysis with Bray-Curtis distance (*r* = 0.47, *p* ≤ 0.001). The pairwise comparisons between habitats using ANOSIM confirmed significant differences (*p* ≤ 0.001, Table S1), indicating that each habitat exhibits its own unique resistome composition. Especially the resistomes of freshwater amphipod guts were profoundly different from other habitats, as supported by high r values between amphipod guts and all other habitats (*r* ≥ 0.77, Table S1). This indicates that a dramatic compositional shift of the resistomes occurred during the low-level trophic level transition from microbiomes of aquatic and benthic food sources to amphipod gut microbiomes. Wastewater and amphipod gut samples spread widely in the ordination plot, indicating higher site-to-site variability compared to the other habitats (Fig. 2b). This was confirmed by showing that distance to centroid was in many pairwise comparisons significantly higher in these habitats (*p* < 0.05 based on Tukey’s HSD test) (Fig. S2). The resistomes of downstream waters were more similar to effluent resistomes than resistomes of any other habitat (Fig. 2b). This effect of wastewater influence was not apparent in any of the other habitats. River water thus appeared to be most significantly impacted by wastewater discharge among all habitats.

**Fig. 3:**
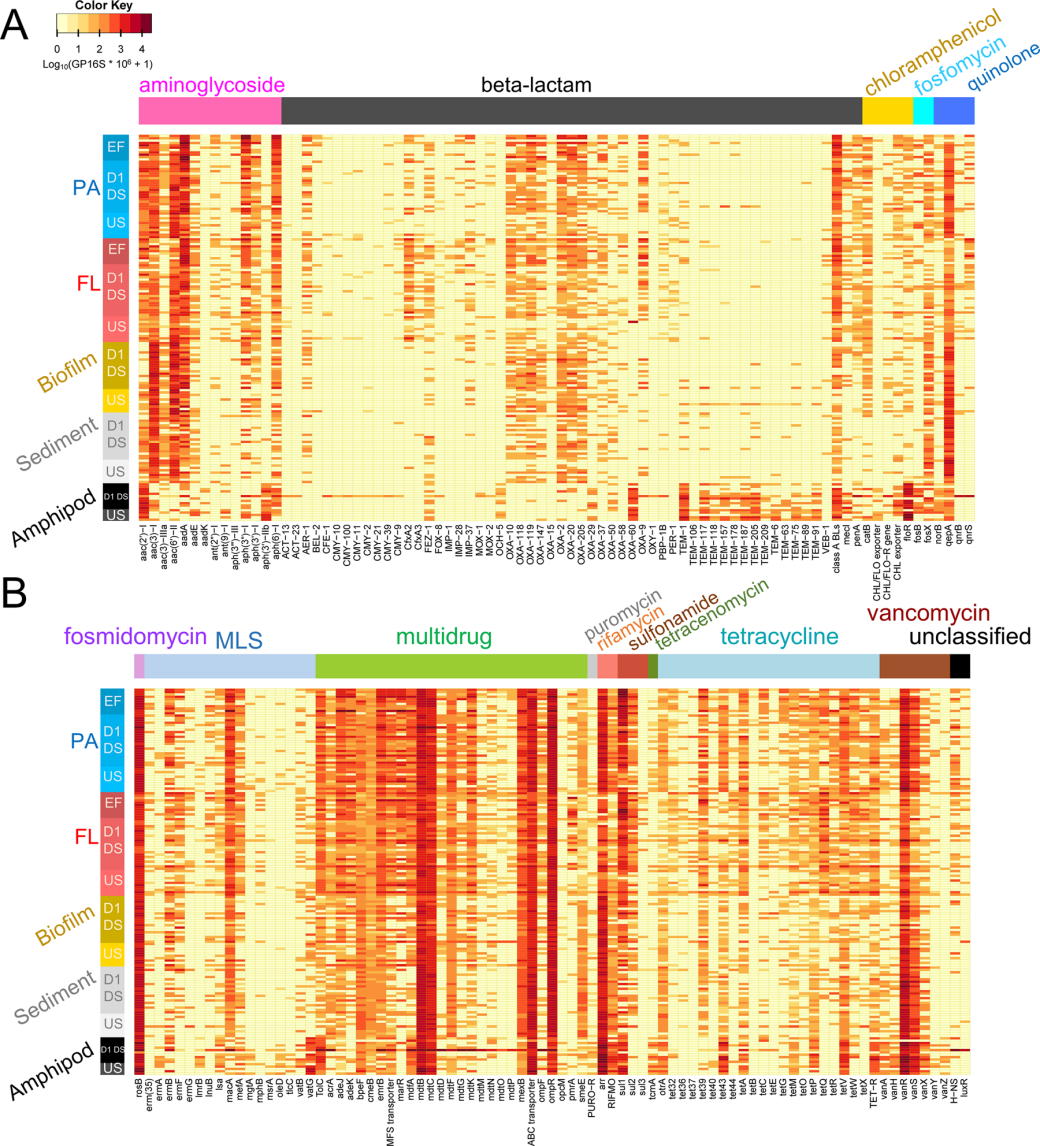
Heatmaps displaying relative abundances of selected ARGs selected based on their explanatory power in ordination (see Fig. 2) in the metagenomes of different river habitats. **A** ARGs conferring aminoglycoside, beta-lactam, chloramphenicol, fosfomycin and fluoroquinolone resistance. **B** ARGs conferring macrolide-lincosamide-streptogramin (MLS), multidrug, sulfonamide, tetracycline and other resistances. Relative abundance was log-transformed (i.e., Log_10_(GP16S × 10^6^ + 1)) for the plot. Each row indicates a sample (arranged by sample type). PA denotes particle-associated (PA, > 5.0 μm) wastewater (EF) or river water biomass from different locations (D1, DS, US) and FL denotes free-living (FL, 0.2–5.0 μm) wastewater or river water biomass. BLs, CHL, FLO, MFS, PURO, RIFMO, and TET stand for beta-lactamase, chloramphenicol, florfenicol, major facilitator superfamily, puromycin, rifampin monooxygenase, and tetracycline, respectively. ‘-R’ indicates ‘-resistance gene’.

Among a total of 677 ARG subtypes identified from 165 samples, we identified 165 ARG subtypes from 15 different resistance classes that were significantly correlated with the ordination (*p* ≤ 0.001 based on permutation test (*n* = 5000)) (Fig. 2b). Those 165 ARG subtypes were visualized as heatmaps showing patterns in ARG abundance and between habitats (Fig. 3). While differences between habitats are apparent, we also note that general patterns of high and low-abundance ARG subypes persist across most or all studied habitats. Some ARGs occurred in high abundances only in amphipod gut samples, notably many TEM genes (e.g. TEM-1/205/117 and the extended spectrum beta-lactamase TEM-118), OXA-60, *aph(3’)-IIb*, *floR*, *vanG*, *arr*, *vanR*, and others. (Fig. 3). Some other ARGs (e.g., *aadA*, OXA-9/10/147, *CfxA2*, *sul1*, and *tet39*/*Q*/*O)* occurred in high abundances in the other habitats, especially for particle-associated and free-living bacteria from effluent samples (Fig. 3). OXA genes occurred differently from TEM genes although both families confer resistance to the same class of antibiotics (i.e., beta-lactams) (Fig. 3a). Many OXA genes have been commonly found in gene cassettes of class 1 integrons from clinical and environmental samples [30–33]. Considering that the gene cassettes of class 1 integrons typically contain multiple resistance genes [34], many bacteria having OXA genes might also possess other ARGs, which could make them multi-resistant. Existence of multiple strong drivers (i.e., various antibiotics) during and/or prior to WWTP stages might select OXA genes over other families (e.g., TEM), which could result in their high relative abundances in effluent water samples. However, the OXA-60 gene occurred with higher abundance in amphipod guts than in the other habitats, and showed a pattern similar to most TEM genes (Fig. 3). OXA-60 is thought to be chromosomal, and not associated with class 1 integrons because there is no core site or inverse core site for recombination which enables the gene to be inserted into a gene cassette [35, 36]. For this reason, OXA-60 might experience different ecological selection processes compared to mobilized OXA genes. However, our study based on short-read-based analysis does not allow to assess co-location between OXA and class 1 integron genes or chromosomes. A future study involving genomic assembly would be required to confirm the abovementioned hypothesis.

**Fig. 4:**
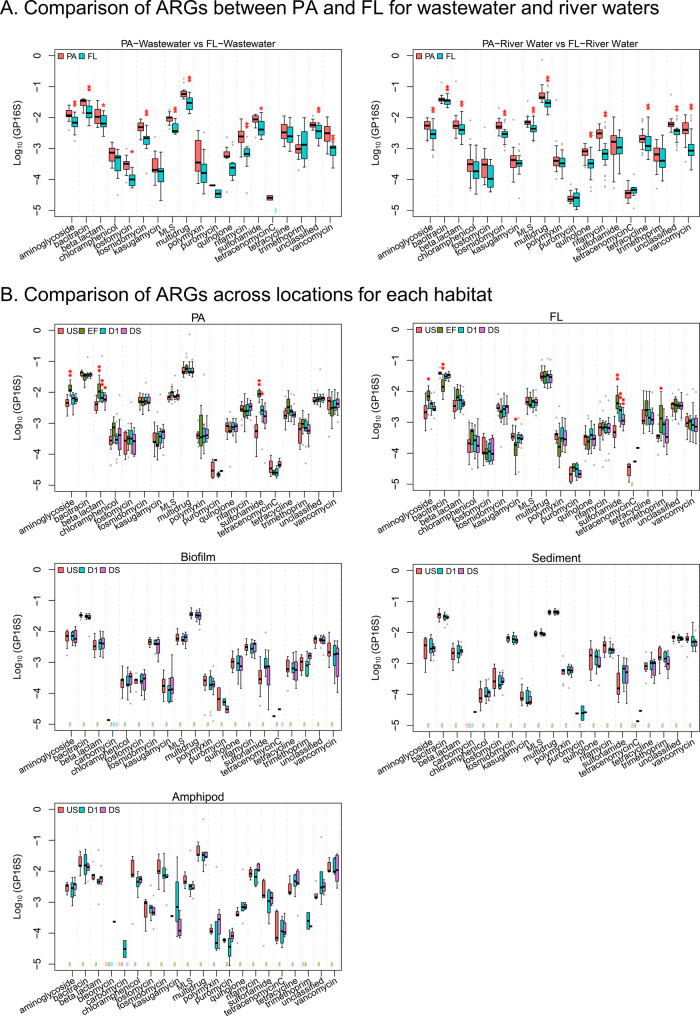
Relative abundances of class-level aggregated ARGs that were identified by metagenomic analysis for each compartment. **A** Comparison between particle-associated (PA) and free-living (FL) biomass from wastewater (EF; left) and river water (US, D1, and DS; right). **B** Occurrences of ARGs in the different habitats: PA, and FL fraction of water, biofilm, sediment, and freshwater amphipod gut. The asterisks (‘*’ indicates *p* < 0.05; ‘**’ indicates *p* < 0.01) indicate significant differences between PA and FL (for **A**), or between US versus the other locations (EF, D1, and DS). MLS indicates macrolide-lincosamide-streptogramin (for **B**). The ARG classes that occurred in at least one of the locations (US, EF, D1, or DS) were shown for each habitat.

The relative abundance of many ARGs (12 out of 19 ARG classes; excluding unclassified ones) was significantly higher in particle-associated than in free-living water bacteria (*p* < 0.05). Those ARG classes included aminoglycoside, bacitracin, beta-lactam, fosfomycin, fosmidomycin, macrolide-lincosamide-streptogramin, multidrug, quinolone, rifamycin, sulfonamide, tetracycline, and vancomycin resistance genes, and other unclassified ARGs (Fig. 4a). We speculate that particle-associated bacteria live in multi-species associations either in aggregates (e.g., flocs or biofilm fragments) or attached to particles. The resulting spatial proximity may increase the levels of chemical (e.g., excretion of antagonistic substance) [37] and/or genetic communications (e.g., horizontal gene transfer) between cells [38], which may result in more active selection and proliferation of ARGs. It has been reported that microbial community composition differs in particle-associated and free-living communities [39, 40]—such differences could also result in different resistomes. Unfortunately, due to low DNA yields for the particle-associated fraction we were unable to test this hypothesis for our samples. This aspect should thus be explored further in future research.

### Resistomes are structurally correlated with microbiomes

**Fig. 5:**
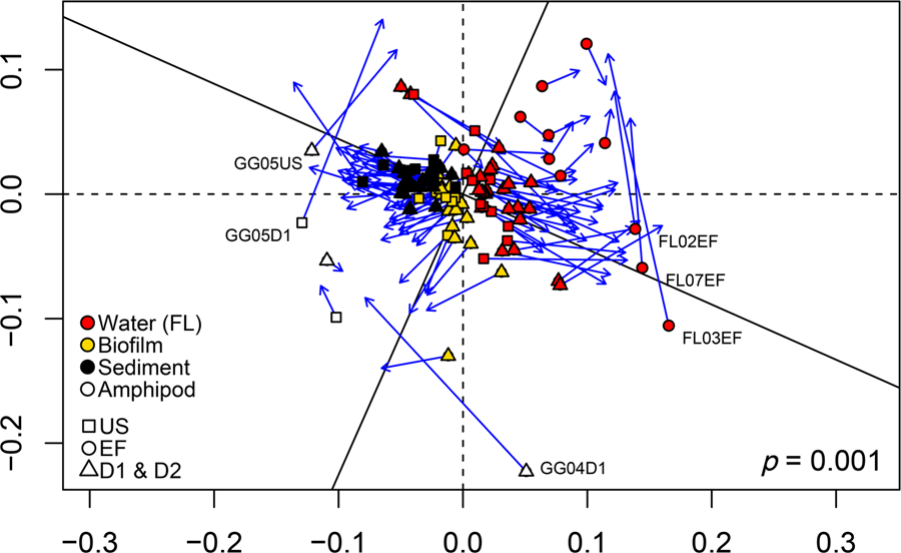
Correlation between resistomes (symbols) and microbiomes (tips of arrows) using Procrustes analysis (*p* = 0.001). Samples with pronounced disagreement between resistome and microbiome structure are labeled (see Dataset S1 for further details on each sample).

To test if bacterial community composition itself could be a key factor driving the differences in resistomes across habitats observed in Fig. 2b, we performed Procrustes analysis to explore the interconnections between resistomes and microbiomes (Fig. 5) (units: GP16S for resistomes, and normalized reads for microbiomes). This dataset included the samples for free-living water bacteria, biofilm, sediment, and amphipod gut samples (see Dataset S1). The *p* value of the Procrustes analysis was significant at 0.1 % level (*p* = 0.001) with Procrustes sum of squares (m12 squared) of 0.5406, and correlation in a symmetric Procrustes rotation of 0.68. This provides strong evidence that resistomes were structurally correlated with microbiomes, confirming one of our initial hypothesis. Indeed, the deviations between each of two datasets (displayed as vector residuals; the longer the vectors the greater the dissimilarity) were in most cases not large (Fig. 5). This result indicates that shifts of microbial communities might be an important driver for changes of resistomes across habitats in riverine environments, largely confirming our initial hypothesis.

Large deviations were noted for some samples. For instance, in three amphipod samples and three samples of the free-living fraction of effluent samples (Fig. 5, labeled icons) the resistome and microbiome structures were decoupled. Thus, changes of microbial communities were not in all cases necessarily related with corresponding changes of ARGs. A profound mobilization of ARGs in those samples, or a local high abundance of clonal bacterial variants with acquired resistances could be possible explanations, but would have to be confirmed by further investigations.

### River water resistomes were significantly impacted by wastewater effluents

Our study sites were selected to have no known point sources of effluents upstream from the studied WWTPs. Accordingly, we did not observe significant signals of pollution at US sites. The effluents from WWTPs significantly increased measures of three physicochemical wastewater indicators (i.e., chloride and sulfate concentrations, and conductivity) in receiving waters (significant differences between US and D1, *p* < 0.05) (Fig. S3). Thus, observed impacts on resistomes downstream of the WWTPs can be assumed to originate mostly from the local WWTP effluents.

A total of 19 resistance classes (excluding bleomycin and carbomycin; see “Methods”, section “Statistics and visualization”) was tested for differences between locations (Wilcoxon signed-rank test). Significant impact (*p* < 0.05) of effluent on D1 (i.e., a significant difference between D1 and US) was observed only in water, and only for some ARG classes, but not in the other habitats (Fig. 4b). The effect was most pronounced for sulfonamide resistance genes for both particle-associated and free-living fractions; for beta-lactam antibiotics resistance genes the difference was significant only for the particle-associated bacteria fraction (Fig. 4b). For aminoglycoside, and trimethoprim resistance genes, relative abundances in effluent were significantly higher (or, for bacitracin and kasugamycin, lower) than at US, but the impact of effluent was not significant at the D1 location.

**Fig. 6:**
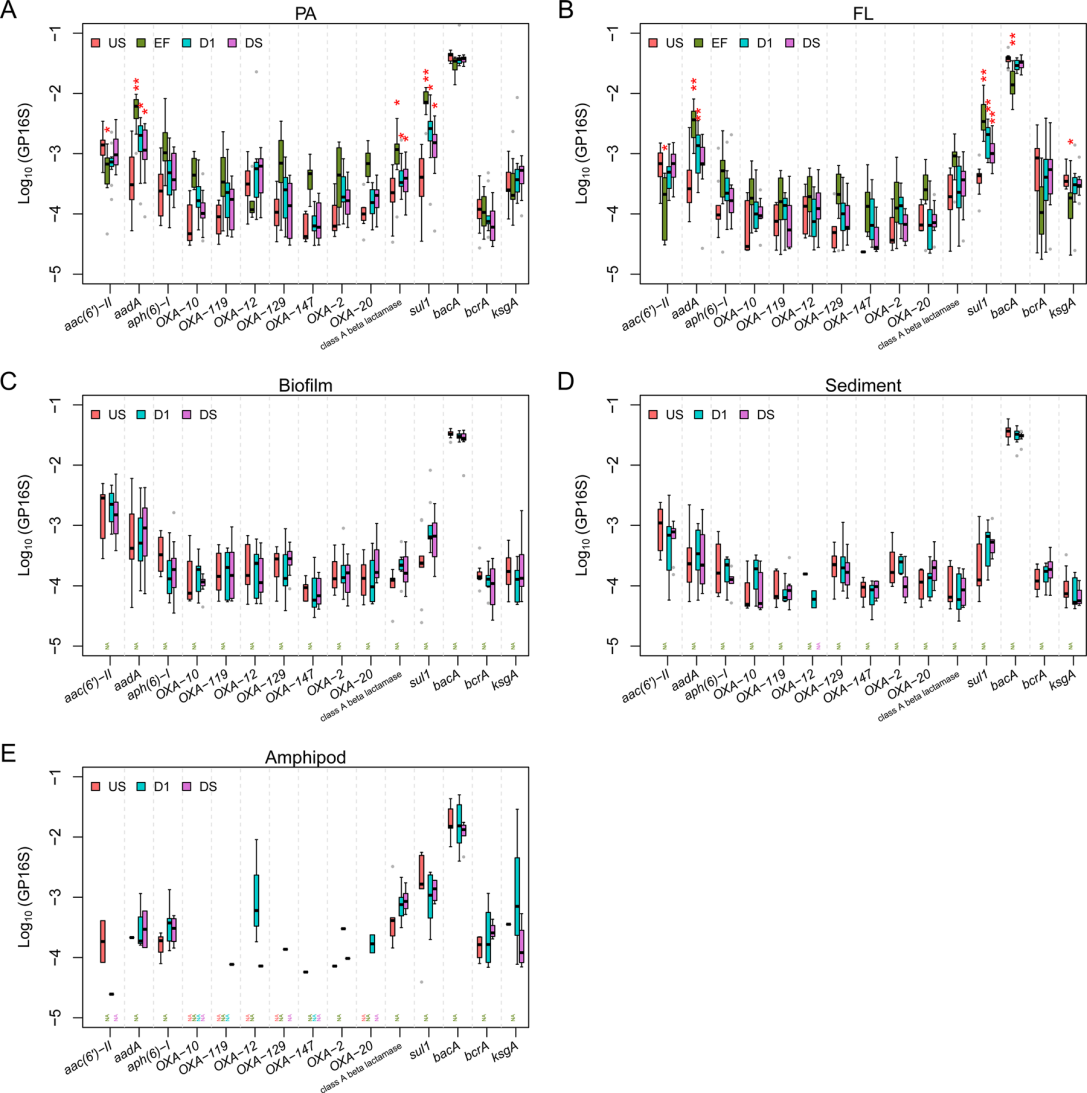
Log_10_-transformed relative abundances of selected (statistically screened; see 3.3) ARGs. **A** particle-associated (PA) river water, **B** free-living (FL) river water, **C** biofilm, **D** sediment, **E** freshwater amphipod gut. The subtypes of aminoglycoside, sulfonamide, and beta-lactam, bacitracin, and kasugamycin resistance genes that show significance differences of relative abundances in location for at least one of the five habitats. The locations (EF, D1, and DS) that are significantly different from US in terms of relative abundances were asterisked (‘*’ indicates *p* < 0.05; ‘**’ indicates *p* < 0.01).

For those classes for which statistically significant impacts were observed (i.e., aminoglycoside, sulfonamide, and beta-lactam resistance for particle-associated bacteria; aminoglycoside, sulfonamide, bacitracin, trimethoprim and kasugamycin resistance for free-living bacteria), we further examined the differences by location for each resistance subtype in the class (a total of 231 subtypes). According to Kruskal–Wallis test, relative abundances of 12 subtypes (*aac(6’)*−*II*, *aadA*, *aph(6)*−*I*, *OXA-2*, *-10*, *-12*, *-20*, *-119*, *-129*, and *-147*, class A beta-lactamase resistance genes, and *sul1*) were significantly different across locations for the particle fraction (*p* < 0.05); 6 subtypes (*aac(6*′*)*−*II*, *aadA*, *bacA*, *bcrA*, *ksgA*, and *sul1*) were significantly different among locations for free-living bacteria (*p* < 0.05); 1 subtype (*OXA-2*) was significantly different among locations for sediment (*p* < 0.05). For the abovementioned 15 subtypes with significant differences (*p* < 0.05; from Kruskal–Wallis test), the post-hoc analysis using Wilcoxon signed-rank test was performed, and the results were shown in Dataset S4, also graphically displayed in Fig. 6. A significant impact of effluent on D1 (i.e., significant differences between US and D1, *p* < 0.05) was observed for some ARG subtypes, for instance, 1 aminoglycoside (*aadA*), 1 sulfonamide (*sul1*), and 1 beta-lactam antibiotic resistance genes (class A beta-lactamase gene) only in particle-associated and/or free-living bacteria (Fig. 6). The relative abundances of those genes (i.e., *aadA*, *sul1*, and class A beta-lactamase gene) were particularly high in effluent compared to US waters, which readily explains why the effects were observed most clearly for these genes. There have been many other studies that previously reported that relative abundances of the aforementioned types of resistance genes are high in effluents [9, 17, 41, 42]. This could be either due to the high inputs of *aadA*, *sul1*, and class A beta-lactamase gene from raw sewages (i.e., untreated wastewaters) [17, 41, 42], or due to their increases during wastewater treatment processes [9]. These results indicate that “co-occurrence” of *aadA*, *sul1*, and class A beta-lactamase gene could be a useful indicator of anthropogenic AMR contamination in river water. Among these genes, *sul1* has already been considered as indicator for anthropogenic pollution by many studies [4, 12, 43, 44].

### No consistent effects of wastewater effluent on non-water habitats

We did not see significant differences of relative abundances of resistance classes between sampling locations in the upstream and downstream for amphipod guts (Fig. 4b) using Kruskal–Wallis test (*p* > 0.05). Evidence of accumulation or enrichment of wastewater-derived ARGs in amphipod guts due to effluent discharges into the rivers were thus not observed in this study (US-D1/DS comparison). We further noted no similarities of amphipod gut resistomes to those of the potential food source, such as biofilm. These results lead us to reject one of our initial hypothesis - wastewater-born ARGs do not appear to be transferred to, accumulated, or enriched in the gut microbiome of low trophic level fauna. However, it should be noted that amphipods are mobile and may have moved between upstream and downstream locations over their lifetime, which could also explain the lack of locational differences. On the other hand, the resistome of the arthropod gut microbiome was unique and enriched for certain ARG (e.g. TEM genes and OXA-60), indicating more studies on the resistomes of aquatic organisms are needed.

There were also no significant differences in the relative abundances of resistance classes of ARGs (a total of 19) between locations for the other habitats (sediments and biofilms) (Fig. 4b). For biofilms, the median relative abundance of sulfonamide resistance genes was increased in the D1 sites compared to US by 55% (Fig. 4b), but the difference between D1 and US was not significant based on the non-parametric Wilcoxon signed-rank test (*p* = 0.07). Similar results were obtained for sediment.

These findings indicated that wastewater inputs did not consistently lead to sweeping and broad changes of the resistomes of the “sessile” downstream bacterial communities in biofilm, sediment or amphipod guts, partially rejecting one of our initial hypothesis. WWTP effluent was not found to affect downstream resistomes in all habitats of the river. Instead, this effect was limited to the water itself.

Our statistical tests mainly indicate that consistent effects over broad resistance classes and across many different sites were difficult to detect in the metagenomics resistomes data. Other studies have shown that effects, sometimes pronounced effects, can often be seen in individual sites [12, 14, 16–18], or using different methodological approaches, such as at our own study sites when using phenotypic screening of AMR bacteria followed by metagenomics [21]. One of the reasons for not finding stronger effects for non-water habitats could be that bacterial cells living in sediments, epilithic biofilms, and amphipod guts in attached-growth forms, are stable over longer periods of time (compared to the water habitats) and form complex, diverse communities with many interdependencies [45–47]. Thus, these communities are subject to ecological and evolutionary processes that may limit their invadability by the wastewater-adapted bacteria in the effluent. However, due to the higher detection limit of metagenomic sequencing compared to, for instance, PCR-based approaches [9], our analysis cannot exclude that such communities are invaded by certain resistant bacteria of wastewater origin (especially low-abundance ones) or receive resistance determinants from wastewater bacteria by horizontal gene transfer. Our results just indicate that, if such effects occur, they do not affect the overall resistomes at the resolution studied here, or that such effects do not occur consistently enough at the study sites to be statistically significant in our analysis.

The lack of strong drivers for resistance selection might also be one of the reasons for limited effects on the “attached growth” habitats. A Swiss-wide project where 12 WWTPs receiving domestic sewage were studied showed that the concentrations of the majority of antibiotics in effluents did not exceed proposed predicted no effect concentrations (PNECs) for resistance selection [9, 48] (Fig. S4). Considering that the concentrations in wastewater are subject to further dilution by up to one order of magnitude after discharge into rivers, concentrations of antibiotics in the receiving waters will be even lower. Thus, the possibilities for antibiotics-mediated resistance selection are very low or absent. Another study performed in two strongly wastewater-impacted Swiss rivers also revealed that concentrations of antibiotics in downstream waters were lower than PNECs [12, 48]. Our current study sites (streams and rivers receiving treated domestic wastewaters) are similar to these sites, so we do not expect high concentrations of antibiotics in receiving waters in this study.

While our results showed that the impact of effluent on the resistomes of non-water habitats is less clear than in the water, this does not mean effluent does not have an impact on downstream non-water habitats in all cases. The degree of impact depends largely on two factors, namely the concentration of contaminants and resistant bacteria in the effluent and the proportion of effluent to river discharge. Repeated samplings at selected rivers with high wastewater inputs might reveal effects on the downstream resistome that were not apparent in this multi-site study. For example, it has been reported that the abundances of ARGs in biofilms and/or sediments increased after receiving wastewaters in some cases, for example in a river receiving untreated or poorly treated wastewaters [18], treated wastewater containing hospital origin contaminants [16], or high volumes of treated wastewaters [12, 14, 17]. These references suggest that at highly contaminated sites a contrast in the resistomes is expected between US and downstream non-water habitats. Our results however suggest that such an effect is non-existent or small for average communal WWTPs discharging into rivers with sufficiently high flow volumes in Switzerland. Follow-up studies to determine the level of contamination that leads to alterations of the resistome will be required to define quality standards for sanitation infrastructure in the context of AMR contamination of aquatic systems.

### Riverine resistomes in the One Health context

Significant alteration of water resistomes due to WWTP effluent and the lack of such effects on resistomes of non-water habitats suggests that “water habitats” could be prioritized when it comes to the surveillance of riverine AMR.

The different riverine microbiomes nevertheless deserve further study. We found certain ARGs in relatively high abundance (e.g., TEM-1/205/117 and OXA-60) in amphipod gut microbiomes. The extent to which these environmental reservoirs contribute to current human infections with resistant pathogens on the one hand or, perhaps more importantly, long-term resistance evolution on the other, cannot yet be answered from our data. A published attribution study from the Netherlands suggests that the contribution of environmental reservoirs to at least some currently circulating antimicrobial resistances of particular clinical concern are low [49]. However, for Switzerland and for many other types of resistance such data are currently not available, and the role of environmental resistance reservoirs for the emergence and evolution of resistance over longer periods of time remains largely unexplored. In the context of systems with (relatively) low levels of contamination, as studied here, additional efforts should be undertaken to study the long-term (evolutionary) impact of chronic exposure of microbiomes in diverse river habitats to AMR, mobile genetic elements, and potential substances with antibiotic resistance selective potential released with WWTP effluents.

### Supplementary information

Supplementary information

Datasets S1-S4

## Data Availability

Raw shotgun sequencing reads are archived in China National GeneBank (CNGB) (Project ID: CNP0002503), and also available in NCBI Sequence Read Archive (SRA) (BioProject: PRJNA844538). Additional datasets not referenced in the manuscript and R scripts used for statistical analysis and graphics are available at the institutional data repository of Eawag (opendata.eawag.ch) at 10.25678/0008PK.
