# Supplementary material for: Differential effects of wastewater treatment plant effluents on the antibiotic resistomes of diverse river habitats
Source: ISME J. 2023 Sep 8;17(11):1993–2002. doi: 10.1038/s41396-023-01506-w (PMC10579368; doi:10.1038/s41396-023-01506-w)
Supplement: Supplementary file 1 — Supplementary information [file 41396_2023_1506_MOESM1_ESM.docx]

**Supplementary Information for :**

**Differential effects of wastewater treatment plant effluents on the antibiotic resistomes of diverse river habitats**

Jangwoo Lee^1,2, +,#^, Feng Ju^3, 4, +, *^, Karin Beck^1^, Helmut Bürgmann^1, *^

^1^Eawag, Swiss Federal Institute of Aquatic Science and Technology, 6047 Kastanienbaum, Switzerland

^2^Department of Environmental Systems Science, ETH Zurich, Swiss Federal Institute of Technology, Zurich, Switzerland

^3^Key Laboratory of Coastal Environment and Resources of Zhejiang Province, School of Engineering, Westlake University, 310024 Hangzhou, Zhejiang, China

^4^ Westlake Laboratory of Life Sciences and Biomedicine, 310030 Hangzhou, Zhejiang, China

^+^These authors equally contributed to this work.

^*^Corresponding author email contact: [helmut.buergmann@eawag.ch](mailto:helmut.buergmann@eawag.ch) (H.B.) and [jufeng@westlake.edu.cn](mailto:jufeng@westlake.edu.cn) (F.J.)

^#^Current address: Departments of Microbiology, Immunology & Infectious Diseases, Cumming School of Medicine, and Biological Sciences, Faculty of Science, University of Calgary, Calgary, Alberta, Canada

**Supplementary Materials**


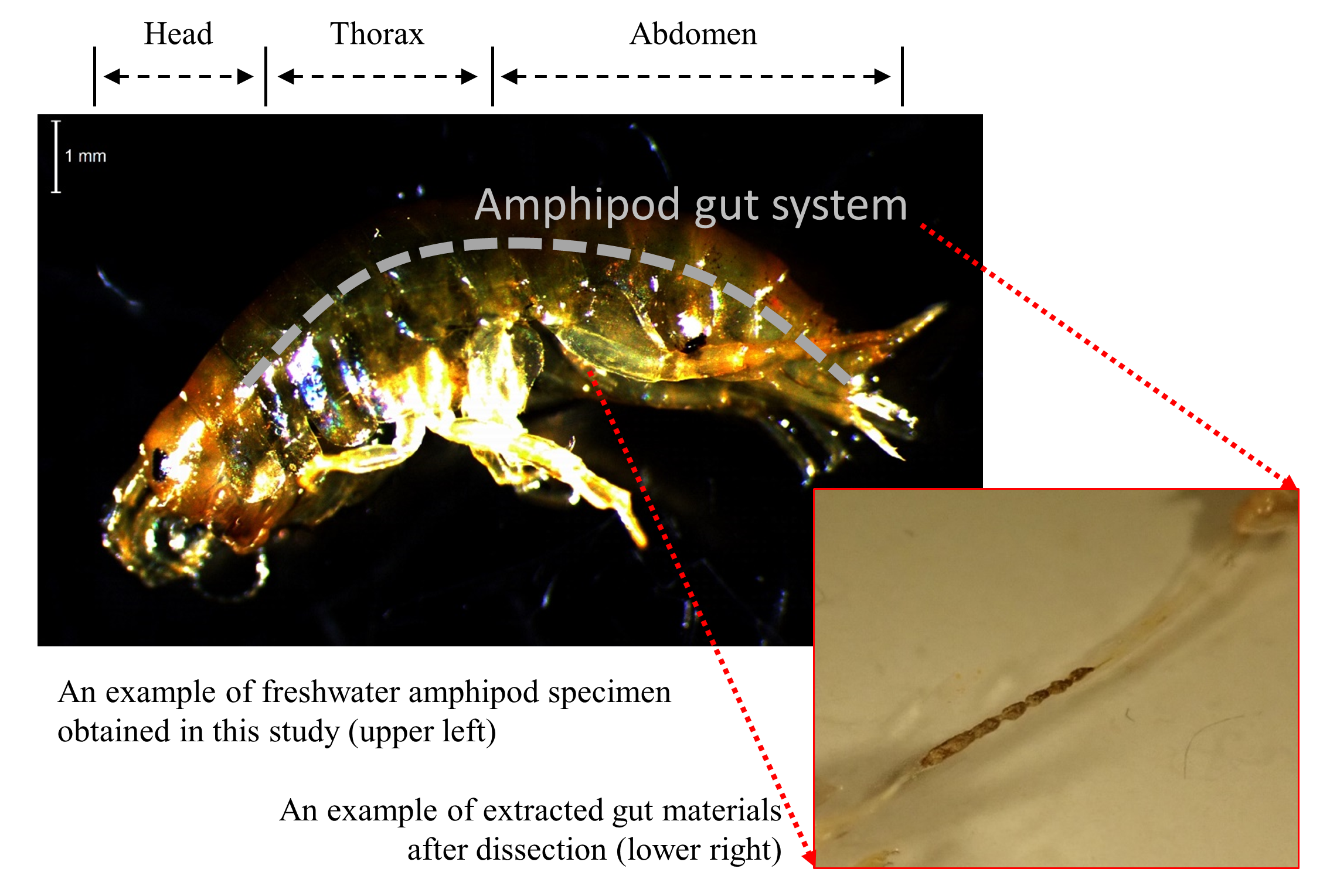


**Figure S1**. An example of freshwater amphipod (i.e., gammarid) specimen and the extracted gut system.


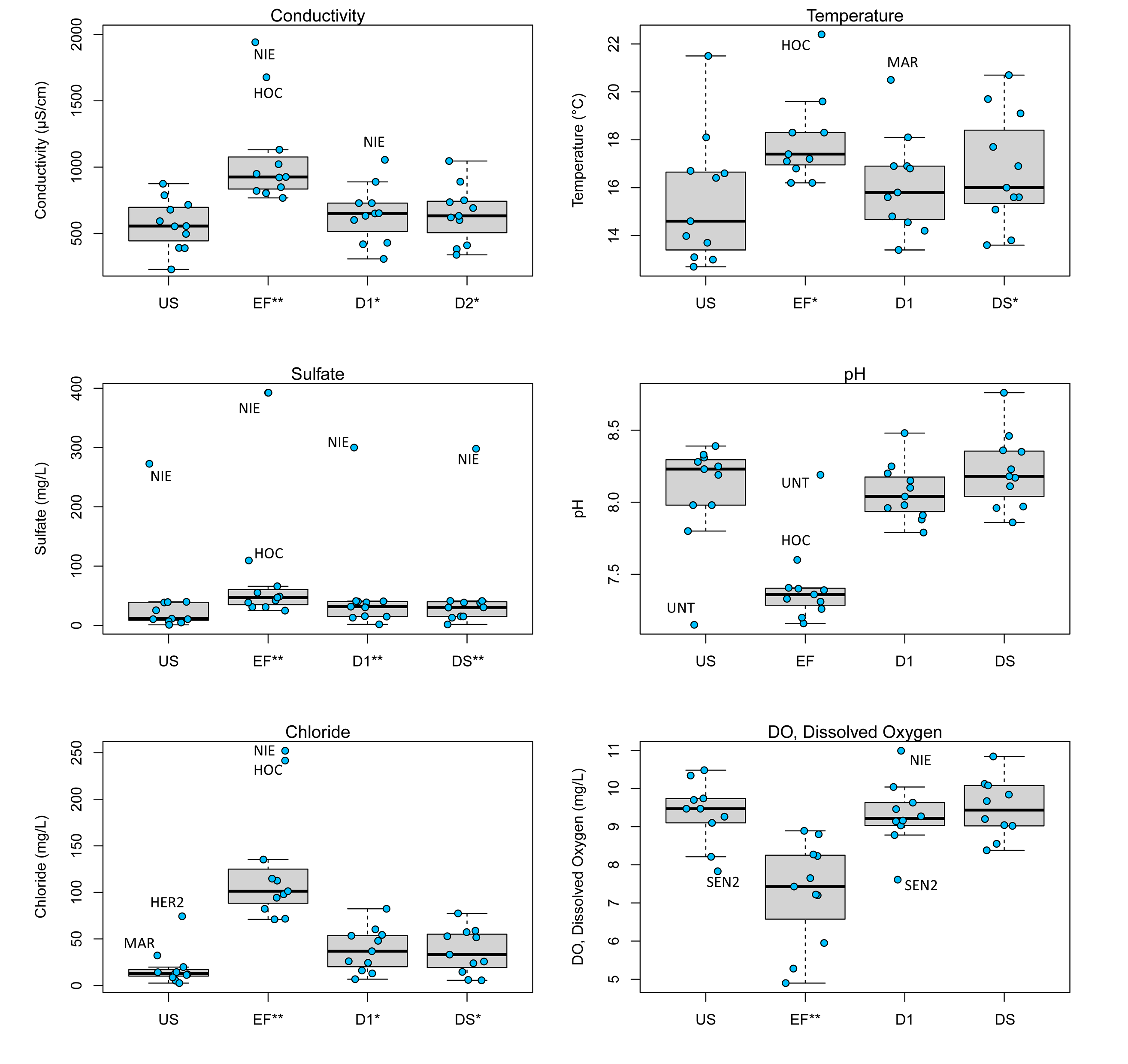


**Figure S2.** Profiles of on-site parameters (sulfate, dissolved oxygen, chloride, temperature, conductivity, and pH) at each sampling location from 11 sampling campaigns. There were significant differences in location (p < 0.05) for all the parameters. Those locations showing significant differences against the upstream (US) location were asterisked. (*) indicates p < 0.05; (**) indicates p < 0.01.


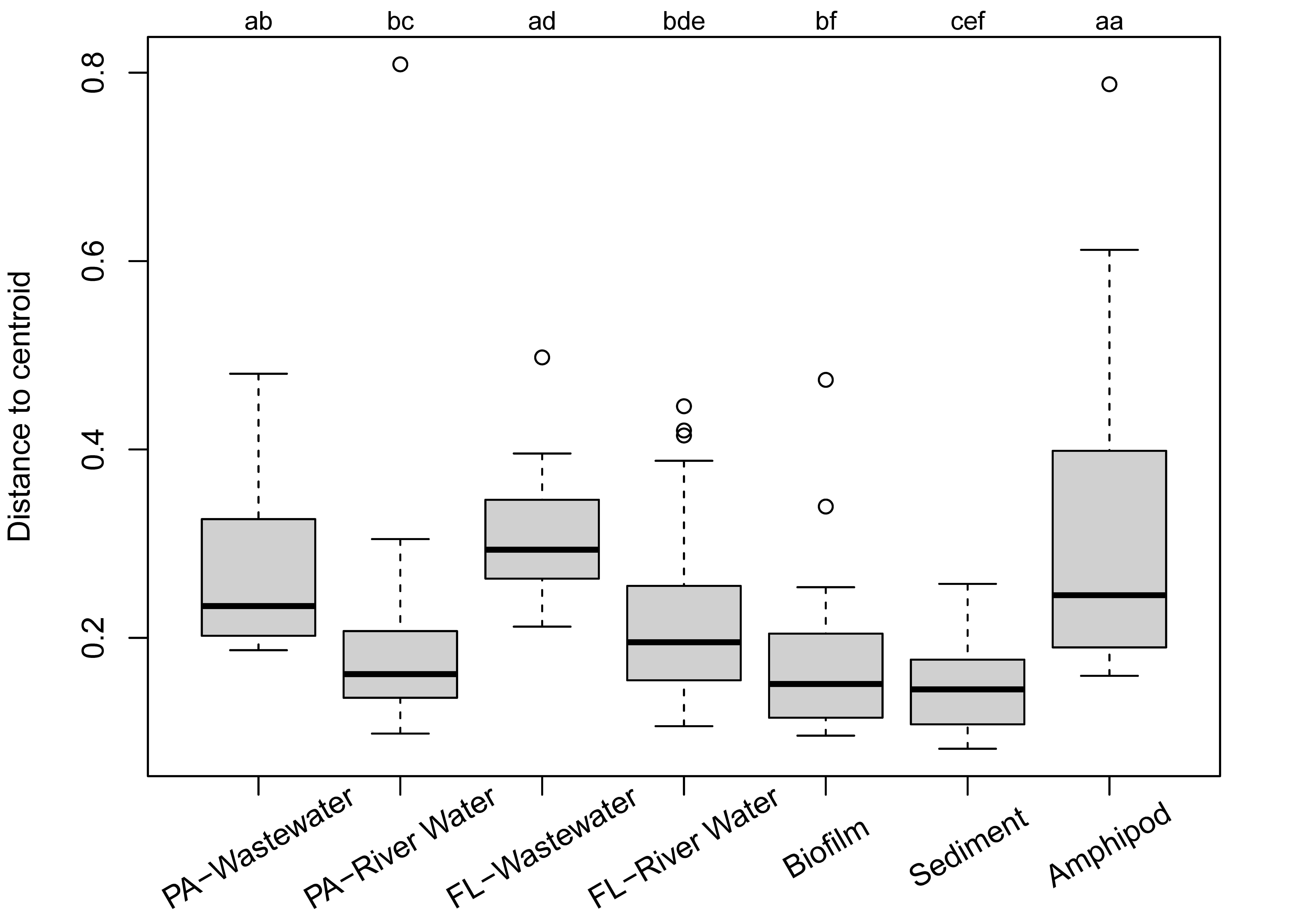


**Figure S3.** Comparison of Bray-Curtis distance from each data point (i.e., each site) to centroid by habitat, providing a measure of variability in the ordination space (Fig. 2b). Pairwise comparison between each of two habitats was performed using Tukey’s HSD test. Those habitats which share the same letter are not statistically different from each other.


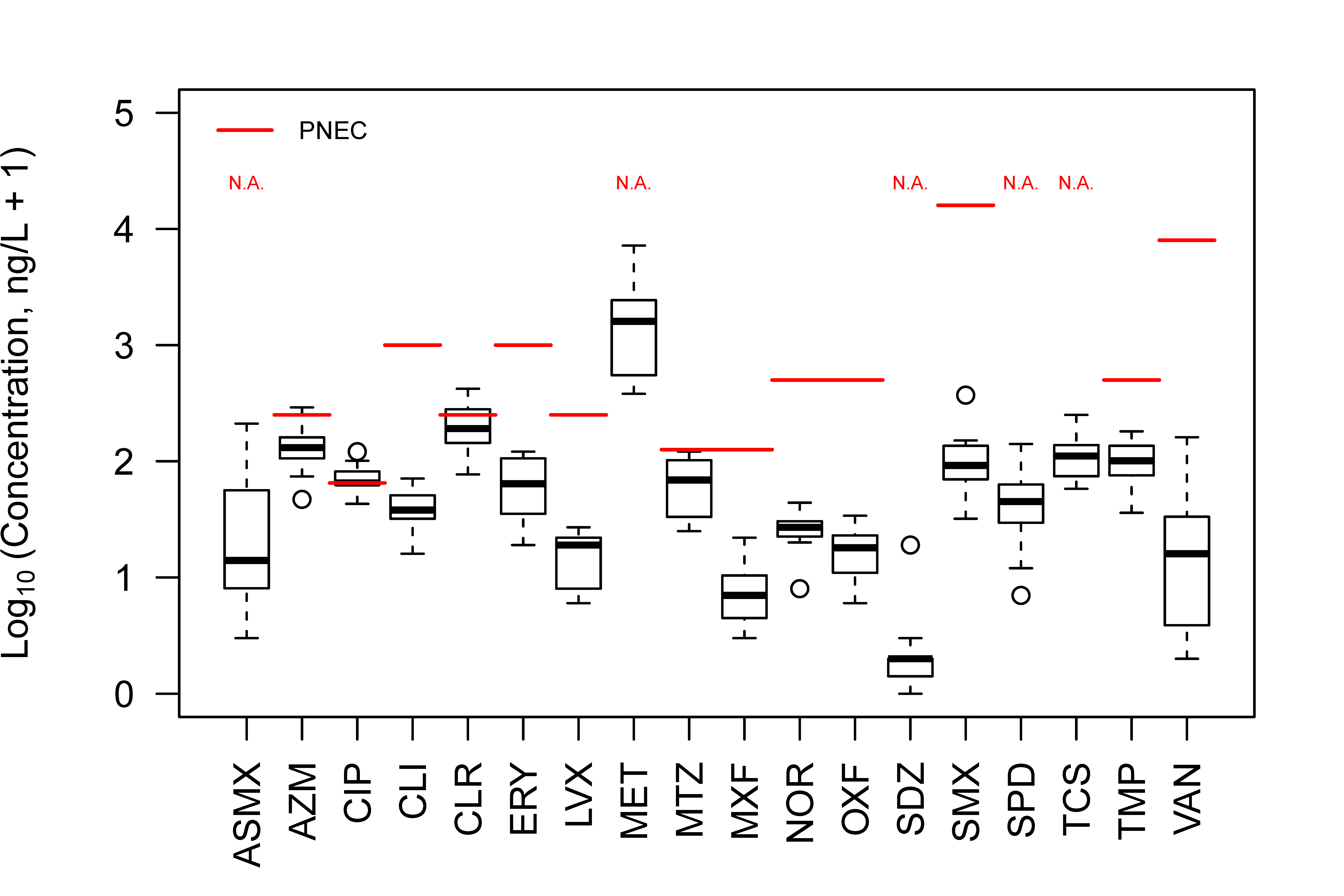


**Figure S4.** Concentrations of different antibiotics and micropollutants in the effluents of 12 wastewater treatment plants in Switzerland (Ju et al., 2019). The predicted no effect concentrations (PNECs) for resistance selection were displayed as red lines (Bengtsson-Palme & Larsson, 2016). N.A. indicates ‘Not available’. ASMX: N4_acetylsulfamethoxazole, AZM: Azithromycin, CIP: Ciprofloxacin, CLI: Clindamycin, CLR: Clarithromycin, ERY: Erythromycin, LVX: Levofloxacin, MET: Metformin, MTZ: Metronidazole, MXF: Moxifloxacin, NOR: Norfloxacin, OXF: Ofloxacin, SDZ: Sulfadiazine, SMX: Sulfamethoxazole, SPD: Sulfapyridine, TCS: Triclosan, TMP: Trimethoprim, VAN: Vancomycin

**Table S1**. Results for pairwise analysis of similarities (ANOSIM) between each of the pairs between river habitats. The p-values were obtained from permutation test (n = 999; the lowest possible value is 0.001). PA indicates particle-associated biomass, FL denotes free-living biomass from river waters. The pairs including amphipod gut show high *r* values (≥0.77), and highlighted in light red.

| **Pair** | ***r*** | **p-value** |
| --- | --- | --- |
| Biofilm-FL | 0.34 | ≤ 0.001 |
| Biofilm-Amphipod Gut | 0.82 | ≤ 0.001 |
| Biofilm-PA | 0.26 | ≤ 0.001 |
| Biofilm-Sediment | 0.36 | ≤ 0.001 |
| FL-Amphipod Gut | 0.84 | ≤ 0.001 |
| FL-PA | 0.31 | ≤ 0.001 |
| FL-Sediment | 0.60 | ≤ 0.001 |
| Amphipod Gut-PA | 0.77 | ≤ 0.001 |
| Amphipod Gut-Sediment | 0.83 | ≤ 0.001 |
| PA-Sediment | 0.43 | ≤ 0.001 |
